# Supplementary material for: Content-rich biological network constructed by mining PubMed abstracts
Source: BMC Bioinformatics. 2004 Oct 8;5:147. doi: 10.1186/1471-2105-5-147 (PMC528731; doi:10.1186/1471-2105-5-147)
Supplement: Additional File 2 — The original results of the above study (non-essential files are deleted to keep the file size under the limit set by BMC bioinformatics). [file 1471-2105-5-147-S2.bz2 › chilibotAdditionalFile2/dip30/332ID8387891E3928/html/TNFRSF1A_LTA.html]

 


 **TNFRSF1A** and **LTA** 
  
Found 102 abstracts in PubMed, retrieved 30.  
 

 What does Google say? 
 PDF only 
| .edu only 

---

**Interactive relationship** (e.g. stimulation, inhibition, etc)

**Neutral relationship**- TNF and LT alpha  [ **LTA** ]  both bind to the tumor necrosis factor receptors TNFR  [ **TNFRSF1A** ] .  Ref: 12163535 J Neurophysiol, 2002
- LT consists of soluble LT alpha  [ **LTA** ]  3, which binds to TNF R1  [ **TNFRSF1A** ]  and TNF R2, and membrane LT alpha  [ **LTA** ]  1 beta 2, which binds to LT beta R.  Ref: 12882833 Int Immunol, 2003
- As the TNF beta  [ **LTA** ]  TNFR1  [ **TNFRSF1A** ]  co crystal structure was the first in the family to be solved, rational drug design based on the ligand receptor interaction sites is more advanced.  Ref: 11467075 Curr Drug Targets, 2000
- TNF alpha and LT alpha  [ **LTA** ]  are both members of the TNF family of molecules and cause cell death by apoptosis following interaction with their counter receptors, the TNFR1  [ **TNFRSF1A** ]  and TNFR2, leading to a cascade of proteolytic events culminating in the blebbing of the cytoplasmic membrane, nuclear condensation and DNA fragmentation.  Ref: 11140465 Arch Immunol Ther Exp Warsz , 2000

**Non-interactive relationship** (e.g. studied together, co-existance, homology, etc.)

- In this review we summarize the essential findings about the function of tumour necrosis factor TNF and its cognate receptors TNFR1  [ **TNFRSF1A** ]  and TNFR2, and lymphotoxin alpha  [ **LTA** ]  LT alpha  [ **LTA** ]  ligands in immune mediated CNS inflammation and demyelination.  Ref: 11004118 Brain, 2000
- Immunity to Toxoplasma gondii critically depends on TNFR  [ **TNFRSF1A** ]  type I mediated immune reactions, but the precise role of the individual ligands of TNFR1  [ **TNFRSF1A** ] , TNF and lymphotoxin alpha  [ **LTA** ]  LTalpha, is still unknown.  Ref: 12794148 J Immunol< MedlineTA>J Immunol, 2000
- This activity of LT depends on membrane LT alpha  [ **LTA** ]  1 beta 2 and not soluble LT alpha  [ **LTA** ]  3, since DA virus persisted and induced inflammatory demyelination in LT beta R, but not TNF R1  [ **TNFRSF1A** ]  or TNF R2, mice.  Ref: 12882833 Int Immunol, 2003
- The crystal structures of TNF alpha, TNF beta  [ **LTA** ] , the extracellular domain of TNFR 1  [ **TNFRSF1A** ]  denoted sTNFR 1, and the TNF beta  [ **LTA** ]  sTNFR 1 complex have been defined by crystallography.  Ref: 10891884 Microsc Res Tech, 2000
- The results from TNFR1  [ **TNFRSF1A** ]  IgG transgenic mice suggest that the hypermetabolic and anorectic responses induced by LPS are independently regulated since, in the absence of TNF alpha or LT alpha  [ **LTA** ] , the LPS induced hypermetabolism is almost prevented but not the anorexia.  Ref: 11125311 Eur Cytokine Netw, 2000
- Use of gene knockout mice indicates that depletion of BM B cells is dependent on TNF alpha, lymphotoxin alpha  [ **LTA** ] , and both TNF receptors, TNFR1  [ **TNFRSF1A** ]  p55 and TNFR2 p75.  Ref: 12444124 J Immunol, 2002
- The effect of SEB to induce EAE in IL 6 mice depends on TNF receptor type 1 TNFR1  [ **TNFRSF1A** ]  signaling because IL 6 TNF LT alpha  [ **LTA** ]  and IL 6 TNFR1  [ **TNFRSF1A** ]  are refractory to SEB.  Ref: 11477542 Eur J Immunol, 2001
- There were significant differences between HAM patients and normal controls in the distributions of TNF promoter region polymophism at position 857, the LT alpha  [ **LTA** ]  gene NcoI polymorphism, and the T G substitution in exon 6 of the TNFR  [ **TNFRSF1A** ]  2 gene.  Ref: 11163081 Hum Immunol, 2000
- To study the role of tumour necrosis factor alpha TNF alpha on some of these metabolic responses to endotoxin, we have used transgenic mice expressing soluble tumour necrosis factor receptor 1 IgG fusion protein TNFR1  [ **TNFRSF1A** ]  IgG as well as TNF alpha knockout KO, lymphotoxin alpha  [ **LTA** ]  LT alpha  [ **LTA** ]  KO, and interferon gamma receptor IFN gamma R KO mice.  Ref: 11125311 Eur Cytokine Netw, 2000
- The heterotrimeric lymphotoxin alpha  [ **LTA** ]  1 beta 2 LTalpha 1 beta 2 complex and LIGHT, a new member of the tumor necrosis factor TNF superfamily, have been identified as membrane anchored ligands for the LTbeta receptor LTbetaR, a member of the TNF receptor TNFR  [ **TNFRSF1A** ]  superfamily.  Ref: 11429164 J Interferon Cytokine Res, 2001
- In contrast, although Th1 cytokine receptors tumor necrosis factor TNF RI  [ **TNFRSF1A** ] , interferon IFN gammaRalpha, IFN gammaRbeta, were detected, their cytokines IFN gamma, TNF alpha, lymphotoxin alpha  [ **LTA** ]  were not.  Ref: 11810184 Acta Neuropathol Berl , 2002
- IL 2 induced tumor necrosis factor TNF beta  [ **LTA** ]  expression further analysis in the IL 2 knockout model, and comparison with TNF alpha, lymphotoxin beta, TNFR1  [ **TNFRSF1A** ]  and TNFR2 modulation.  Ref: 11157847 Int Immunol, 2001
- We studied tumor necrosis factor TNF, lymphotoxin alpha  [ **LTA** ]  LT alpha  [ **LTA** ] , and TNF receptors type 1 TNFR 1  [ **TNFRSF1A** ]  and type 2 TNFR  [ **TNFRSF1A** ]  2 gene polymorphisms as well as HLA class II DRB1 alleles in Japanese patients with human T cell lymphotropic virus type I HTLV I associated myelopathy HAM n = 51, patients with adult T cell leukemia lymphoma ATL n = 48, asymptomatic HTLV I carriers n = 50, and HTLV I seronegative, normal controls n = 112.  Ref: 11163081 Hum Immunol, 2000
- Results demonstrated that rejection of the B16.MUC1 tumor cell line was primarily mediated by CD4 T cells, and required Fas ligand, lymphotoxin alpha  [ **LTA** ] , CD40, CD40 ligand and CD28, but not perforin, gammadelta T cells, IL 4, IL 10, IL 12 or tumor necrosis factor receptor 1  [ **TNFRSF1A** ] .  Ref: 12147624 Int Immunol, 2002
- The serum concentrations of tumor necrosis factor beta  [ **LTA** ]  TNF beta  [ **LTA** ] , TNF receptor 1 TNFR 1  [ **TNFRSF1A** ] .  Ref: 11939943 Acta Neurol Scand, 2002
- Surprisingly, both perforin and FasL played unique roles during the effector phase of immunity to Panc02.MUC1, while lymphotoxin alpha  [ **LTA** ] , but not TNFR 1  [ **TNFRSF1A** ] , was required for immunity against Panc02.MUC1 tumors.  Ref: 12111121 Cancer Immunol Immunother, 2002
- The results suggest that the TNF LT alpha  [ **LTA** ]  gene region within the HLA class III of chromosome 6 and the TNFR  [ **TNFRSF1A** ]  2 gene region located on chromosome 1p36 might contribute to susceptibility to HAM,.  Ref: 11163081 Hum Immunol, 2000
- The different expression patterns of the two distinct TNFR  [ **TNFRSF1A** ]  homologues may be critical in determining whether binding with TNF alpha or TNF beta  [ **LTA** ]  have activating, proliferative, or apoptotic effects on target cells.  Ref: 12631519 Dev Comp Immunol, 2003
